# HIPK2 modulates p53 activity towards pro-apoptotic transcription

**DOI:** 10.1186/1476-4598-8-85

**Published:** 2009-10-14

**Authors:** Rosa Puca, Lavinia Nardinocchi, Ada Sacchi, Gideon Rechavi, David Givol, Gabriella D'Orazi

**Affiliations:** 1 Department of Experimental Oncology, Molecular Oncogenesis Laboratory, National Cancer Institute "Regina Elena", Rome, Italy; 2 Department of Oncology and Neurosciences, University "G D'Annunzio", Chieti, Italy; 3 Cancer Research Center, Chaim Sheba Medical Center, Tel-Hashomer and Sackler School of Medicine, Tel-Aviv University, Tel-Aviv, Israel; 4 Department of Molecular Cell Biology, Wiezmann Institute of Science, Rehovot, Israel

## Abstract

**Background:**

Activation of p53-mediated gene transcription is a critical cellular response to DNA damage and involves a phosphorylation-acetylation cascade of p53. The discovery of differences in the response to different agents raises the question whether some of the p53 oncosuppressor functions might be exerted by different posttranslational modifications. Stress-induced homeodomain-interacting protein kinase-2 (HIPK2) phosphorylates p53 at serine-46 (Ser46) for p53 apoptotic activity; p53 acetylation at different C-terminus lysines including p300-mediated lysine-382 (Lys382) is also required for full activation of p53 transcriptional activity. The purpose of the current study was to evaluate the interplay among HIPK2, p300, and p53 in p53 acetylation and apoptotic transcriptional activity in response to drug by using siRNA interference, p300 overexpression or deacetylase inhibitors, in cancer cells.

**Results:**

Knockdown of HIPK2 inhibited both adriamycin-induced Ser46 phosphorylation and Lys382 acetylation in p53 protein; however, while combination of ADR and zinc restored Ser46 phosphorylation it did not recover Lys382 acetylation. Chromatin immunoprecipitation studies showed that HIPK2 was required *in vivo *for efficient p300/p53 co-recruitment onto apoptotic promoters and that both p53 modifications at Ser46 and Lys382 were necessary for p53 apoptotic transcription. Thus, p53Lys382 acetylation in HIPK2 knockdown as well as p53 apoptotic activity in response to drug could be rescued by p300 overexpression. Similar effect was obtained with the Sirt1-inhibitor nicotinamide. Interestingly trichostatin A (TSA), the inhibitor of histone deacetylase complexes (HDAC) did not have effect, suggesting that Sirt1 was the deacetylase involved in p53 deacetylation in HIPK2 knockdown.

**Conclusion:**

These results reveal a novel role for HIPK2 in activating p53 apoptotic transcription. Our results indicate that HIPK2 may regulate the balance between p53 acetylation and deacetylation, by stimulating on one hand co-recruitment of p300 and p53Lys382 on apoptotic promoters and on the other hand by inhibiting Sirt1 deacetylase activity. We attempted to reactivate p53 apoptotic transcriptional activity by rescuing both Ser46 and Lys382 modification in response to drug. Our data propose combination strategies for the treatment of tumors with dysfunctional p53 and/or HIPK2 that include classical chemotherapy with pharmacological or natural agents such as Sirt1-deacetylase inhibitors or zinc, respectively.

## Background

The tumor suppressor p53 plays a critical role in the prevention of human cancer and in tumor response to chemotherapy. As a transcription factor that both activates and represses target genes p53 demands a highly complicated network to control and fine-tune responses to the different stress-signals encountered [1]. Stress-induced modifications of p53 variously implicated in protein stability and/or transcriptional activity include phosphorylation, acetylation, and ubiquitylation, as well as conformational changes and interactions with other proteins [2]. Much interest was lately given on what specific p53 posttranslational modification can affect specific p53 oncosuppressor outcome. It has been proposed that Ser46 phosphorylation is a late event after DNA damage that triggers irreversible apoptosis by shifting p53 from cell-cycle-related to apoptosis-related gene transcription (e.g., p53AIP1 gene) [3,4]. We have previously shown that homeodomain-interacting protein kinase-2 (HIPK2) phosphorylates p53 at N-terminal Ser46 enhancing p53 apoptotic [5]. Thus, HIPK2-induced Ser46 phosphorylation activates several p53 targets involved in both intrinsic and extrinsic apoptotic pathway [5-7]. However, Ser46 phosphorylation is not always sufficient to induce apoptosis in all cell types suggesting that multiple mechanisms of regulation of p53 might exist [8].

Full activation of p53 transcriptional function involves also p53 acetylation by coactivators/histones acetyl-transferases (HATs) that occurs specifically in the C-terminal regulatory regions surrounding the tetramerization domain [9-11] and facilitates the recruitment of HATs to p53 target promoters [12]. Phosphorylation of p53 N-terminal residues permits the interaction of p53 with CBP/p300, which acetylates p53 lysine-382 (Lys382) and with PCAF, which acetylates p53 lysine-320 [13]. This is followed by an increase in p53 stability and sequence-specific DNA-binding activity, both *in vitro *and *in vivo*, possibly due to conformational changes [9,11,13,14]. It has been shown that HIPK2 and CBP/p300 show a mutual interaction and that HIPK2-mediated phosphorylation of p53Ser46 is required for the CBP-mediated p53 acetylation [15]. Moreover, HIPK2 interacts with p300 and HIPK2-mediated phosphorylation of p300 stimulates its acetyl-transferase (HAT) activity [16]. These data suggest a complex interplay among HIPK2, p300, and p53 and indicate that HIPK2 may act at multiple levels to fine-tune transcriptional activity of p53 in tumor cells subjected to genotix stress.

A tight regulation of p53 acetylation *in vivo *implies also the involvement of deacetylases [13]. In this regard, it has been shown that Lys382 of p53 is a substrate for the Sirt1 (NAD-dependent histone deacetylase)-mediated deacetylation which antagonizes p53-dependent transcriptional activation and apoptosis in response to DNA damage and oxidative stress [17,18]. Sirt1-deficient mice display increased levels of radiation-induced apoptosis and p53 hyperacetylation [19]. On the other hand, p53 can repress Sirt1 transcription as shown by *Tp53*-null mice with increased levels of Sirt1 in various tissue types as well as by several p53-null tumor cells lines with Sirt1 overexpression [20,21]. These findings underline the role of p53 transcription activity for a proper Sirt1/p53 regulatory feedback loop to induce p53 oncosuppressor function.

We reported previously that HIPK2 knockdown by RNA interference results in p53 protein misfolding with ablation of p53 transcriptional activity and that zinc restores wtp53 native conformation, DNA binding, and transcriptional activity [22]. Unravelling the modes of p53 transcriptional selection is a key factor in the understanding of how p53 is able to choose life or death for the cell. In this report, in order to understand how different p53 posttranslational modifications and proteins interactions are involved in influencing p53 promoter selection we evaluated the role of HIPK2 in Ser46 and Lys382 modifications for p53 apoptotic transcription. We found that combination of ADR and zinc in HIPK2 knockdown cells restored Ser46 phosphorylation but it did not recover Lys382 acetylation. We found that p300 and p53Lys382 were co-recruited onto apoptotic promoters and that both Ser46 phosphorylation and Lys382 acetylation were necessary for efficient p53 apoptotic transcriptional activity in response to drug. Lack of Lys382 acetylation in HIPK2 knockdown did not depend on p300 downmodulation but could rather depend on impaired p53/p300 interaction, as suggested by the ChIP analysis and/or on increased deacetylase activity, as suggested by increased Sirt1 levels. Lys382 acetylation in HIPK2 knockdown and p53 apoptotic transcriptional activity could be restored in response to drug by p300 overexpression or by the use of the Sirt1 physiological inhibitor nicotinamide in combination with drug and zinc.

Our results show that p53Ser46 phosphorylation could be obtained in response to drug even in the absence of HIPK2, while HIPK2 knockdown strongly impaired Lys382 acetylation and consequently p53 apoptotic transcriptional activity. As p53 apoptotic function is often deregulated in tumors by different mechanisms, our results indicate that low HIPK2 expression is a potential marker for p53 dysfunction, tumor progressions, and chemoresistance; they also suggest some strategies, combining chemotherapy with pharmacological or natural agents, aimed at restoring p53 apoptotic transcriptional function in tumors with non-functional wtp53 and/or HIPK2.

## Results

### Lack of p53Lys382 acetylation impairs in vivo p53 binding to apoptotic gene promoters when HIPK2 is underexpressed

**Figure 1:**
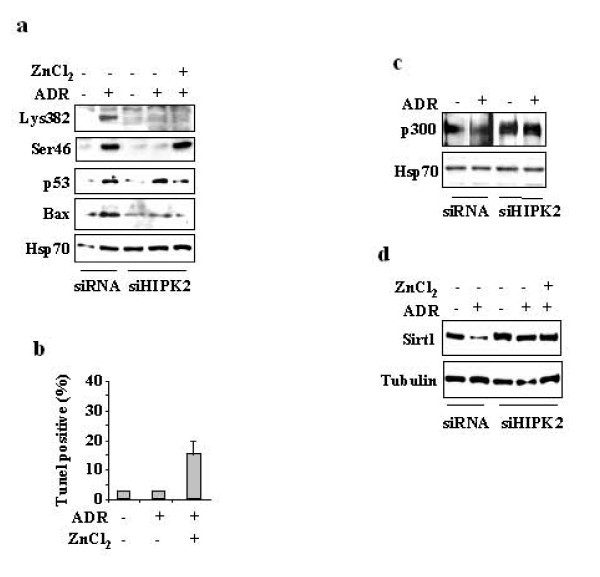
**Analysis of p53Lys382 acetylation, p53Ser46 phosphorylation, Bax, p300 and Sirt1 protein levels, in HIPK2 knockdown in response to ADR and zinc treatment**. (**a**) RKO cells transfected with pSuper-HIPK2 (siHIPK2) or pSuper control (siRNA C) for 24 h were treated with ADR (2 μg/ml) and ZnCl_2 _(150 μM) for 16 h. After treatment, equal amounts of total cell extracts were subjected to Western immunoblotting using specific antibodies as indicated. Anti-Hsp70 was used as protein loading control. (**b**) RKO cell depleted of HIPK2 function by siRNA were treated with ADR and zinc and 24 h and apoptosis was measured by TUNEL assay and shown as percentage of TUNEL positive cells. The results shown are representative of two independent experiments performed in duplicate, ± S.D. (**c**) Equal amount of nuclear cell extracts of RKO cells depleted of HIPK2 function as in (a) and treated with ADR (2 μg/ml) for 16 h were analysed by Western immunoblotting with anti-p300 antibody. Anti-Hsp70 was used as protein loading control. (**d**) Equal amount of total cell extracts of RKO-HIPK2-depleted cells as in (a) and treated with ADR (2 μg/ml) for 16 h were analysed by Western immunoblotting with anti-Sirt1 antibody. Anti-tubulin was used as protein loading control.

We recently reported that zinc supplementation to ADR-treated HIPK2 depleted cells (siHIPK2) shifts the misfolded p53 protein into wild-type conformation, restoring p53 DNA binding and transactivation function as well as Ser46 phosphorylation [22,23]. To gain insight into the re-establishment of the p53 pathways when HIPK2 is underexpressed Ser46 phosphorylation and Lys382 acetylation were first analysed by Western immunoblotting. As shown in Figure 1a, knockdown of HIPK2 (siHIPK2), obtained with pSuper-HIPK2 transfection (not shown), inhibited both ADR-induced Ser46 phosphorylation, and Lys382 acetylation; however, while combination of ADR and zinc restored Ser46 phosphorylation it did not recover Lys382 acetylation (ac-p53Lys382). Interestingly, ADR and zinc combination did not induce Bax expression in HIPK2 depleted cells (Figure 1a), compared to control cells, although it induced a good level of apoptosis, as assessed by TUNEL assay (Figure 1b), raising the question whether lack of Lys382 acetylation by HIPK2 depletion could compromise p53 apoptotic response. Lack of ac-p53Lys382 in response to combination of ADR and zinc did not depend on p300 protein downmodulation as p300 protein levels were similarly expressed in siRNA control and siHIPK2 cells (Figure 1b). In addition, Western immunoblotting showed that Sirt1 levels were downmodulated by ADR in control cells while they were not affected by ADR alone or in combination with zinc in HIPK2-depleted cells (Figure 1c). These findings suggest that zinc, likely by reconstitution of wtp53 conformation as previously reported by us [22], allowed ADR-induced Ser46 phosphorylation in siHIPK2 cells possibly by other kinases known to phosphorylate Ser46 after ADR treatment [24]. They also suggest that HIPK2 was required to mediate p53Lys382 acetylation by p300 or by suppressing Sirt1 deacetylase activity.

**Figure 2:**
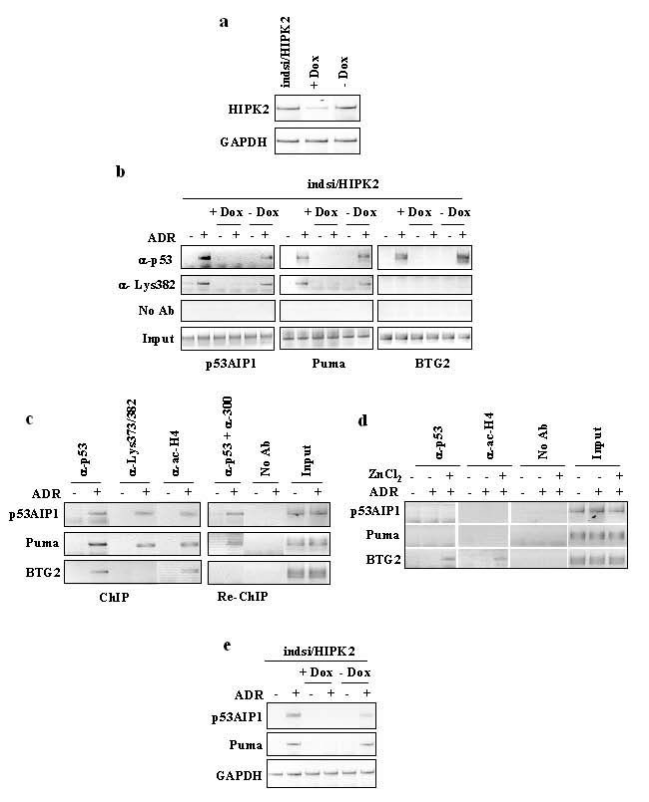
**HIPK2 stimulates co-recruitment of p53Lys382 and p300 on apoptotic promoters**. (**a**) MCF7/indsi/HIPK2 cells were treated with Dox (+Dox) for 5 days (for HIPK2 knockdown) and then cultured without Dox (-Dox) for additional 5 days (for HIPK2 recover) before analysing HIPK2 expression by RT-PCR. GAPDH was used as a control of efficiency for RNA extraction and transcription. (**b**) ChIP analysis with antibodies specific for p53 and p53 acetylated on Lys382 was performed with MCF7indsi/HIPK2 cells treated with Dox for 5 days and then cultured without Dox for additional 5 days, with or without ADR (2 μg/ml) treatment for 16 h. PCR analyses were performed on the immunoprecipitated DNA samples using specific primers for p53 target promoters, as indicated. (**c**) ChIP analysis with antibodies specific for p53, p53 acetylated on Lys373/382 and PAN-acetylated Histone H4 (ac-H4) was performed with RKO cells, transfected with siRNA control, left untreated or treated with ADR (2 μg/ml) for 16 h. Re-ChIP analysis was performed by immunoprecipitating the p53-containing complexes with an antibody against p300. PCR analysis was performed on the immunoprecipitated DNA samples using specific primers for p53 target promoters. A sample representing linear amplification of the total input chromatin (Input) was included as control. Additional controls included immunoprecipitation performed with non-specific immunoglobulins (No Ab). (**d**) ChIP analysis with antibody specific for p53 and PAN-acetylated Histone H4 (ac-H4) was performed with RKO cells stable depleted of HIPK2 function by pSuper-HIPK2 transfection (siHIPK2) and treated with ADR (2 μg/ml) and zinc (150 μM) for 16 h. PCR analysis was performed on the immunoprecipitated DNA samples using specific primers for p53 target promoters. A sample representing linear amplification of the total input chromatin (Input) was included as control. Additional controls included immunoprecipitation performed with non-specific immunoglobulins (No Ab). (**e**) MCF7indsi/HIPK2 cells were treated as in (b) and induction of p53 target genes *p53AIP1 *and *Puma *was evaluated by semiquantitative RT-PCR, with *GAPDH *serving as control.

To test these hypotheses we determined the requirement of HIPK2 in p53/p300 co-recruitment and Lys382 acetylation for p53 binding to apoptotic promoters and the physiological relevance for apoptotic transcriptional activity by ChIP, luciferase assays, and RT-PCR analysis. We took advantage of the inducible HIPK2 knockdown in the MCF7-indsi/HIPK2 cell line [23] that allows a reversible HIPK2 depletion by doxycyclin (+Dox) treatment and HIPK2 reconstitution after Dox removal (-Dox) from cell culture medium. A representation of HIPK2 mRNA expression after Dox treatment (+Dox) and after Dox removal (-Dox) was shown by reverse-transcriptase (RT)-PCR analysis (Figure 2a); those conditions of HIPK2 depletion and reconstitution were then used for the following experiments. Chromatin immunoprecipitation with or without Dox (and therefore in the absence or presence of endogenous HIPK2, respectively), in the presence or absence of ADR treatment, was carried out with anti-p53 and anti-ac-p53Lys382 antibodies. PCR analysis showed efficient p53 recruitment in response to ADR onto (apoptotic) *p53AIP1*, *Puma*, and (growth-control) *BTG2 *gene promoters in HIPK2 wild-type cells. However the ac-p53Lys382 was recruited only onto *p53AIP1 *and *Puma *promoters (Figure 2b). Following HIPK2 depletion (+Dox), p53 was no longer bound to the apoptotic promoters while the binding was re-established after Dox removal (-Dox) (Figure 2b); again, after HIPK2 reconstitution (-Dox) ac-p53Lys382 was recruited only onto apoptotic promoters (Figure 2b), suggesting an involvement of p300 in ac-p53Lys382 binding to the apoptotic promoters. To verify this hypothesis, ChIP and Re-ChIP experiment was performed. Chromatin complexes of RKO cells transfected with siRNA control and treated with ADR were immunoprecipitated with anti-p53, anti-ac-p53Lys373&382, and anti-PAN-acetylated Histone H4 (ac-H4) antibodies and PCR analysis was performed using specific primers flanking the p53 sequence-specific promoter regions. We found that p53 was efficiently recruited *in vivo*, in response to ADR, onto *p53AIP1*, *Puma*, and *BTG2 *gene promoters, which also increased H4 histone acetylation suggesting an open chromatin structure of the tested promoters (Figure 2c, ChIP panel); notably, p53 immunoprecipitated with anti-ac-Lys373/382 antibody was recruited only onto apoptotic gene promoters *p53AIP1 *and *Puma *(Figure 2c, ChIP panel) suggesting an involvement of p300 in p53 binding to apoptotic promoters. Therefore, Re-ChIP analysis was performed by immunoprecipitating the p53-containing complexes with an antibody against p300 such that only those DNA sequences that are simultaneously bound by both proteins would be amplified by the PCR. As shown in Figure 2c (Re-ChIP, panel), p53 and p300 were co-recruited only onto the apoptotic gene promoters *p53AIP1 *and *Puma *where the ac-Lys373/382 was recruited. Conversely, ChIP analysis performed in siHIPK2 cells showed that p53 and ac-H4 were readily recruited only onto *BTG2 *promoter following zinc supplementation to ADR treatment while they were not recruited onto apoptotic promoters *p53AIP1 *and *Puma *(Figure 2d). In agreement, Re-ChIP analysis performed in siHIPK2 cells failed to show p53 and p300 binding to promoters (data not shown). Remarkably, apoptotic gene transcription *in vivo *of *p53AIP1 *and *Puma *genes was obtained only in the presence of HIPK2, after ADR treatment (Figure 2e). These results suggest that HIPK2 is involved *in vivo *in Lys382 acetylation and in p53/p300 co-recruitment onto apoptotic gene promoters and that Ser46 phosphorylation, as rescued by ADR and zinc combination (Figure 1a), was not sufficient to induce p53 binding to apoptotic promoters when p300 co-recruitment and Lys382 acetylation of p53 were impaired in HIPK2-depleted cells.

### HIPK2 influences p53 apoptotic gene transcription depending on p53 site-specific acetylation

**Figure 3:**
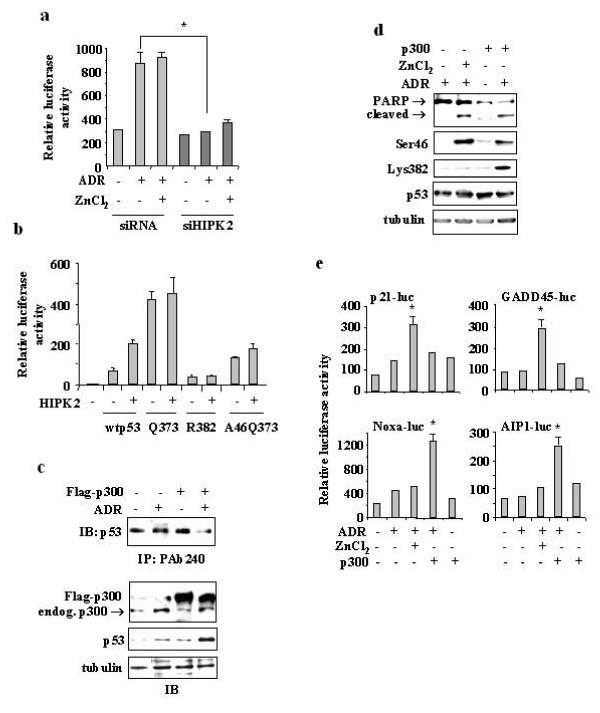
**Ectopic expression of p300 in HIPK2 knockdown rescues the p53Lys382 apoptotic transcriptional activity**. (**a**) RKO cells stable-transfected with p53AIP1-luc reporter were transfected with pSuper-HIPK2 (siHIPK2) or control pSuper (siRNA) vectors for 24 h and then treated with ADR (2 μg/ml) and zinc (150 μM) for 16 h before luciferase activity was assayed. Results are representative of three independent experiments performed in duplicate ± S.D. * *P *< 0.01. (**b**) H1299 cells were co-transfected with p53AIP1-luc reporter and HIPK2-Flag together with wtp53, Q373, R382, or A46Q373 mutants. Luciferase activity was assayed 36 h thereafter. (**c**) RKO cells stable depleted of HIPK2 function were transfected with p300-Flag expression vector and treated with ADR for 16 h. Equal amount of total cell extracts were immunoprecipitated with conformation-specific PAb240 (for p53 mutant, unfolded conformation) antibody and immunoblotted (IB) with anti-p53 (FL393) antibody (upper panel). Endogenous p300 (arrow) and ectopic p300-Flag protein levels were detected by immunoblotting (IB) equal amount of total cell extracts with anti-p300 antibody (lower panel), endogenous p53 levels were also detected. Anti-tubulin was used as protein loading control (lower panel). (**d**) RKO cells stable depleted of HIPK2 function were transfected with p300-Flag expression vector and treated with ADR and zinc for 16 h. Equal amount of total cell extracts were immunoblotted with anti-phospho-Ser46, anti-acetyl-Lys382, anti-p53, and anti-PARP (arrows indicate the uncleaved and cleaved forms) antibodies. (**e**) RKO cells stable depleted of HIPK2 function were co-transfected with the indicated reporters along with p300 expression vectors for 24 h and then treated with ADR for 16 h before luciferase activity was assayed. Results, normalized to β-galactosidase activity, are representative of three independent experiments performed in duplicate ± S.D. * *P *< 0.01.

Here we found that HIPK2 is involved also in Lys382 acetylation that affected p53 binding to apoptotic promoters. We then aimed to assess the functional relevance of the above observations. We have previously demonstrated that RKO undergoes apoptosis and that HIPK2 depletion inhibits p53-dependent apoptosis [6,22] (Additional file 1, Table S1). To gain further insight into the re-establishment of the p53 pathways, we compared gene expression pattern of RKO-siHIPK2 cells treated with ADR and zinc, by cDNA micorarray analyses. We found that treatment with ADR and zinc combination did not restore the p53 apoptotic gene transcription (data not shown). Then we tested the apoptotic transcriptional activity of p53 on p53AIP1-luciferase reporter and found that p53 in siHIPK2 cells was highly impaired in inducing p53AIP1-luc activity in response to ADR, compared to siRNA control cells, even after zinc supplementation (Figure 3a), confirming that Ser46 phosphorylation alone without Lys382 acetylation was not sufficient to induce p53 apoptotic transcriptional activity of the luciferase reporter analysed. To assess the impact of HIPK2-induced p53 posttranslational modifications on p53 specific activation of proapoptotic gene promoters the transcriptional activity of wtp53 compared to that of p53 mutants following HIPK2 overexpression was evaluated in a p53/null cell line (H1299). Both wtp53 and Q373 mutant (mimicking constitutive acetylation of residues 370, 372, 373) induced Noxa-luc reporter activity that was further increased by HIPK2 (Figure 3b), that likely favoured also Lys382 acetylation other than inducing Ser46 phosphorylation, while the nonacetylatable K382R mutant did not induce Noxa-luc activity even after HIPK2 overexpression. Remarkably, mutation of Ser46 to alanine strongly reduced the Q373-mediated Noxa-luc activity that obviously could not be increased by HIPK2 (Figure 3b). Similar results were obtained with p53AIP1-luc reporter (data not shown). These results indicate that HIPK2-dependent p53 apoptotic transcriptional activity is fully functioning only when p53 is posttranslational modified at both Ser46 and Lys382 residues.

### Ectopic expression of p300 rescues the p53 apoptotic gene transcription impaired by HIPK2 depletion

**Figure 4:**
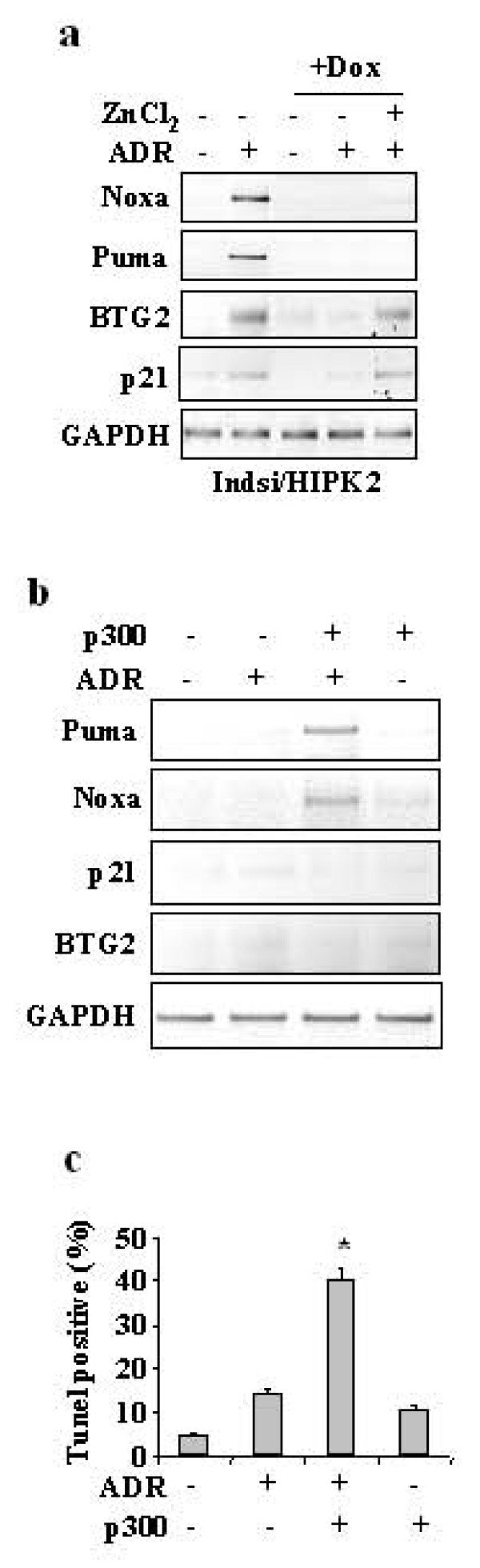
**Ectopic expression of p300 in HIPK2 knockdown rescues the p53 apoptotic gene transcription**. (**a**) MCF7indsi/HIPK2 cells, untreated or treated with Dox for HIPK2 depletion and reconstitution (see Figure 2) were treated with ADR (2 μg/ml) and zinc (150 μM) for 16 h. Gene expression of p53 targets was analysed by semiquantitative RT-PCR. GAPDH was used as a control for efficiency of RNA extraction and transcription. (**b**) RKO cells stable depleted of HIPK2 function were transfected with p300 and treated with ADR and zinc for 16 h. Gene expression of p53 targets was analysed by semiquantitative RT-PCR. GAPDH was used as control. (**c**) RKO cell depleted of HIPK2 function by siRNA were transfected with p300 or with empty vector by using LipofectaminePlus. After transfection cells were trypsinized and replated and 24 h after transfection treated with ADR. Apoptosis was measured by TUNEL assay 24 h after ADR treatment and shown as percentage of TUNEL positive cells. The results shown are representative of two independent experiments performed in duplicate, ± S.D.

We hypothesized that HIPK2 might affect multiple p53 posttranslational modifications, in response to genotoxic stress, to efficiently induce p53 apoptotic target gene transcription. In particular, as acetylation of p53 C-terminal lysines at position 373 and 382 by p300 stimulates these activities [12,25,26], we tested whether ectopic p300 was able to restore p53 apoptotic transcriptional activity in siHIPK2 cells. We first analysed p53 conformation by immunoprecipitation with conformation-specific antibody, as we showed that in the absence of HIPK2 p53 increases its mutant-like conformation that affects its DNA-binding and transcriptional activities [22]. As shown in Figure 3c, overexpression of p300 in siHIPK2 cells reduced the levels of unfolded (mutant-like) p53 conformation as detected by PAb240 antibody. We therefore reasoned that, by modifying the conformation of p53 in response to genotoxic stress, p300 could specifically induce ADR-mediated Ser46 phosphorylation (likely by additional kinases other than HIPK2) in addition to promoting Lys382 acetylation, even in HIPK2 knockdown background, and efficiently saturate low-affinity proapoptotic promoters. Indeed, overexpression of p300 in siHIPK2 cells induced both Lys382 acetylation and Ser46 phosphorylation in response to drug, reactivating the apoptotic response, as assessed by PARP cleavage (Figure 3d). In support of our hypothesis, we performed luciferase assay, in the absence of HIPK2, upon co-transfection of p300 with two p53-induced reporters related to growth arrest (p21-luc and GADD45-luc) and two reporters related to apoptotic function (Noxa-luc and p53AIP1-luc). ADR treatment did not induce luciferase activities of any reporter analysed while zinc supplementation to ADR treatment selectively induced the p21-luc and GADD45-luc reporters (Figure 3e), as expected. Notably, p300 overexpression in siHIPK2 cells specifically induced apoptotic gene reporters in response to drug (Figure 3e). Finally, *in vivo *transcription of p53-apoptotic target genes in response to ADR, greatly impaired in siHIPK2 cells (+Dox) compared to control cells, was not reactivated by zinc (Figure 4a); conversely, p21 and BTG2 gene expression was induced by ADR and zinc combination, as expected (Figure 4a). Of note, p300 overexpression reactivated p53 apoptotic transcription in response to ADR (Figure 4b), while it did not induce p21 and BTG2 gene expression (Figure 4b). In agreement, p300 overexpression in siHIPK2 cells significantly reactivated the ADR-induced apoptotic response, as assessed by TUNEL assay (Figure 4c). These results suggest that forced p53 acetylation in HIPK2-depleted cells can modify wild-p53 conformation such that overcomes the inability of endogenous p53 to activate apoptotic response to drug. This is also in agreement with the role of HIPK2 in inducing both Ser46 and ac-Lys382 posttranslational modifications for p53 apoptotic gene transcription (Figure 3b). In agreement with our results, it has been proposed that various p53 acetylation mutants possess different intrinsic affinities for their downstream promoters; thus covalent modifications of p53 may change target gene preference by imposing conformational changes in p53 that encourage selective recognition of different p53 responsive elements [27]. Particularly, acetylation of residues around position K382 is necessary for binding to low-affinity proapoptotic promoters, it affects the ability to interact with cellular proteins, including p300, and influences other key posttranslational modifications, such as Ser46 [27]. How HIPK2 knockdown can affect p300-dependent p53Lys382 acetylation has not been clearly elucidated yet, although previous studies have shown HIPK2/p300 interaction and that HIPK2-mediated phosphorylation of p300 stimulates its acetyl-transferase (HAT) activity [16].

### Inhibition of Sirt1 with nicotinamide correlates with restored p53 apoptotic transcriptional activity in HIPK2 knockdown

**Figure 5:**
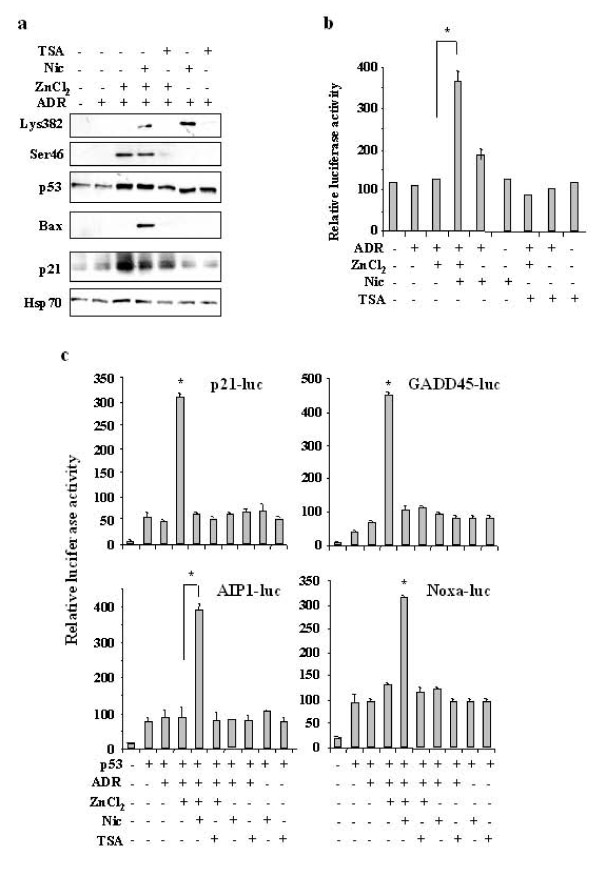
**Nicotinamide reconstitutes p53Lys382 acetylation and apoptotic transcriptional activity**. (**a**) RKO cells stable depleted of HIPK2 function were treated with ADR (2 μg/ml) and zinc (150 μM) for 16 h in the presence of nicotinamide (Nic) or TSA. Equal amounts of nuclear extracts were analysed by Western immunoblotting of Lys382, Ser46, total p53, p21 and Bax. Hsp70 represents protein loading control. (**b**) RKO cells stable depleted of HIPK2 function were transfected with p53AIP1-luc reporter for 24 h and treated with ADR and zinc for 16 h in the presence of Nic or TSA before luciferase activity was assayed. Results, normalized to β-galactosidase activity, are representative of three independent experiments performed in duplicate ± S.D. * *P *< 0.01. (**c**) H1299 cells stable depleted of HIPK2 function were co-transfected with low amount of wtp53 expression vector and p21-luc, GADD45-luc, p53AIP1-luc, and Noxa-luc reporters and 24 h after transfection treated with ADR and zinc for 16 h in the presence of Nic or TSA before luciferase activity was assayed. Results, normalized to β-galactosidase activity, are representative of three independent experiments performed in duplicate ± S.D. * *P *< 0.01.

We hypothesized that HIPK2 may affect p53 apoptotic activity by acting on structural rearrangements of p53 not only through Ser46 phosphorylation but also by exposing p53 C-terminus to p300 and therefore promoting acetylation and transcriptional function of p53 toward apoptotic genes. However, since Lys382 is also a substrate of Sirt1 deacetylase whose regulation has not yet been fully elucidated in tumorigenesis, we attempted to evaluate whether lack of ac-p53Lys382 in our HIPK2 knockdown system but could depend on increased deacetylase activity. To verify this hypothesis, HIPK2-depleted cells were treated with deacetylase inhibitors followed by Western immunoblotting and luciferase assays. As shown in Figure 5a, both Ser46 phosphorylation and Lys382 acetylation were efficiently obtained after combination of zinc and ADR with nicotinamide (Nic), a physiologic inhibitor of Sirt1 deacetylase while trichostain A (TSA), the inhibitor of histone deacetylase complexes (HDAC) did not affect these modifications, indicating that Sirt1 is the potential deacetylase involved. Moreover, while p21 expression was induced after ADR and zinc combination, Bax expression was induced only after adding nicotinamide to ADR and zinc combination (Figure 5a). Luciferase assay in RKO-HIPK2 depleted cells showed that Nic strongly induced p53AIP1-luc reporter activity in combination with zinc and ADR (Figure 5b), whereas TSA did not. Similar results were obtained with H1299-HIPK2-depleted cells where p53 was co-transfected with two reporters related to growth arrest (p21-luc and GADD45-luc) and two reporters related to apoptotic function (Noxa-luc and p53AIP1-luc) and treated with combination of ADR and zinc in the presence of Nic or TSA inhibitors. As shown in Figure 5c, strong p53-dependent induction of p21-luc and GADD45-luciferase activity was obtained only after combination of ADR and zinc, while Nic or TSA did not have any relevant effect; on the contrary, p53-dependent induction of p53AIP1-luc and Noxa-luc activity was strongly induced by adding Nic to ADR and zinc combination treatment, while TSA did not have effect. These data suggest that Sirt1 is the enzyme responsible for p53 deacetylation in our model and that p53 acetylation is necessary for p53 apoptotic transcriptional activity, indeed, Nic to ADR and zinc combination treatment shifted the p53 affinity versus the apoptotic reporters.

**Figure 6:**
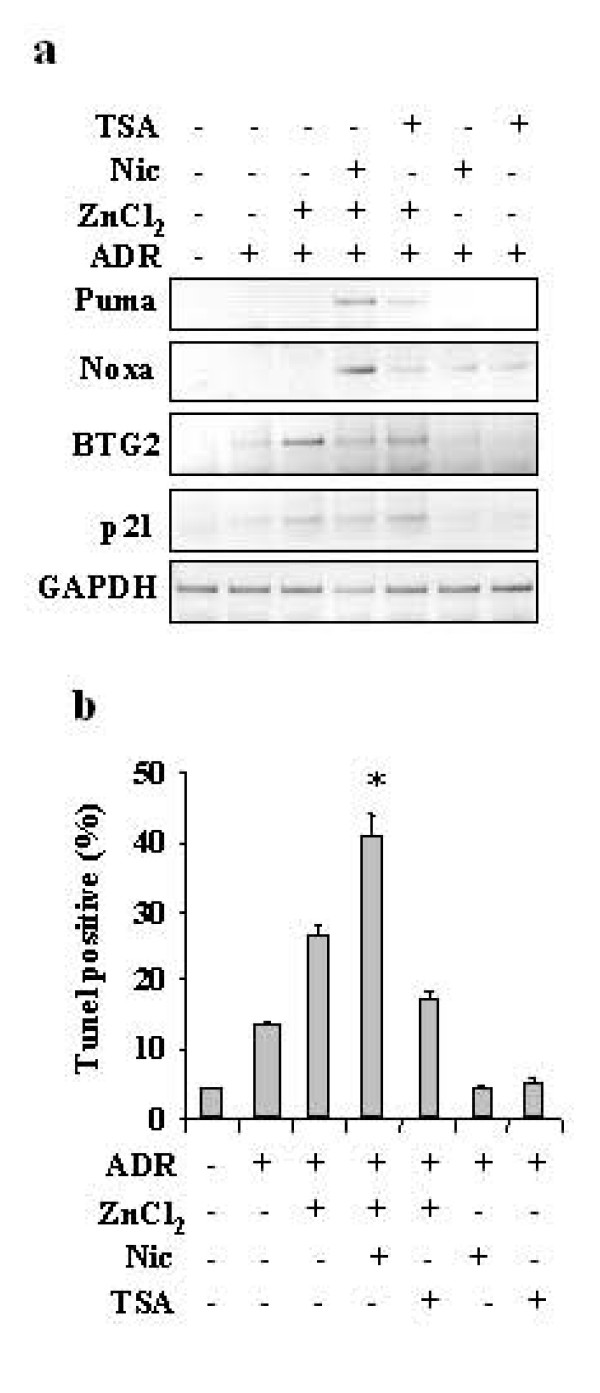
**(a) RKO cells stable depleted of HIPK2 function (siHIPK2) were treated with ADR and zinc for 16 h in the presence of Nic or TSA**. Gene expression of p53 targets was analysed by semiquantitative RT-PCR. GAPDH was used as a control for RNA extraction and transcription. (**b**) RKO cells HIPK2-depleted were treated as in (a) and apoptosis was measured by TUNEL assay 24 h after treatments and shown as percentage of TUNEL positive cells. The results shown are representative of two independent experiments performed in duplicate, ± S.D.

The *in vivo *gene transcription and apoptosis were ultimately measured by RT-PCR analysis and TUNEL assay. As shown in Figure 6a, ADR in combination with zinc induced p21 and BTG2 expression, while only addition of nicotinamide to zinc supplementation strongly induced *Puma *and *Noxa *mRNA expression in response to ADR, in HIPK2-depleted cells, while TSA did not have relevant effect. In agreement, addition of nicotinamide to ADR and zinc combination significantly increased apoptotic response of HIPK2 depleted cells, as assessd by Tunel assay, while TSA did not have relevant effect (Figure 6b). Taken together, these results indicate that the Sirt1-inhibitor nicotinamide can restore p53Lys382 acetylation in HIPK2 depleted cells, and induce p53 apoptotic gene transcription in response to ADR and zinc treatment improving the apoptotic response. These results suggest that HIPK2 or HIPK2-regulated pathways may affect Sirt1 activity and therefore that HIPK2 might regulate the acetylation/deacetylation balance of p53 through likely independent pathways.

## Discussion

In this paper we studied the interconnection between two posttranslational modifications involved in p53 activation, that is Ser46 phosphorylation and Lys382 acetylation. We show that HIPK2 was needed for co-recruitment of ac-p53 and p300 onto apoptotic gene promoters, in response to drug, and that p53 acetylation at Lys382 was required for successful interaction of p53 with its target apoptotic promoters and for improving apoptotic response. Furthermore, for the first time we show here that HIPK2 may also regulate Sirt1 activity.

The best understood functions of p53 have been attributed to its transcriptional activity that undergoes different levels of regulation mainly through posttranslational modifications and protein-protein-interactions, in response to DNA damage [28]. Previous data have indicated that, upon severe DNA damage, phosphorylation of Ser46 specifically favours transactivation of proapoptotic genes [3] and we reported that HIPK2 is one of the kinases that phosphorylate p53 at Ser46, thereby contributing to the increased likelihood of cell death under severe DNA damage [5]. Indeed, HIPK2 depletion by siRNA strongly impairs p53 function, affecting not only Ser46 phosphorylation [6] but also p53 wild-type conformation due to deregulation of metallothionein and zinc [22,23]. In this report, we have further investigated the p53 regulation exerted by HIPK2 and highlighted a complex interplay between HIPK2, p300, Sirt1 and p53 connecting HIPK2-dependent Ser46 phosphorylation with Lys382 acetylation/deacetylation at which HIPK2 might fine-tune the apoptotic transcriptional activity of p53 in tumor cells subjected to genotoxic stress. One important finding that supports the interplay among HIPK2, p300 and p53 comes from a leukemogenesis model where the oncogene CBFβ-SMMHC attenuates the p53 apoptotic function after DNA damage and prevents p300 phosphorylation by sequestering HIPK2 in the cytoplasm [29,30] which becomes the mechanistic explanation of the attenuation of the HIPK2/p53 apoptotic pathway in response to DNA damage. Intriguingly, our finding that also Sirt1 deacetylase may be induced in HIPK2 knockdown highlighted the existence of additional pathways regulated directly or indirectly by HIPK2 that need to be further elucidated. Sirt1 levels are increased in a number of tumor types although only few mechanisms of Sirt1 regulation have been described [31] highlighting the requirement for further deciphering the genetic alterations that have occurred during tumorigenesis that may affect Sirt1. Furthermore, targeting of Sirt1 in tumors overexpressing the protein might be advantageous specially when used in combination with other compounds that target the p53 pathway and this is currently an intense area of investigation.

Covalent modifications of p53 may change target gene preference by imposing conformational changes in p53 that encourage selective recognition of different p53 responsive elements [27]. Thus, various p53 acetylation mutants possess different intrinsic affinities for their downstream promoters. Particularly, acetylation of residues around position K373 is necessary for binding to low-affinity proapoptotic promoters, it affects the ability to interact with cellular proteins, including p300, and influences other key posttranslational modifications, such as Ser46 and Ser392 [27]. Notably, we show that HIPK2 influences p53 apoptotic gene transcription depending on p53 site-specific acetylation as we observed more efficient Noxa- and p53AIP1-luc activity of wtp53 and Q373 mutant, co-expressed with HIPK2 compared to the K382R mutant that failed to induce luciferase activity (Figure 3b). These results imply that both posttranslational modifications of p53, Ser46 phosphorylation and Lys382 acetylation, are required for full activation of apoptotic transcription, as suggested by the analysed p53 apoptotic target genes.

The most common mechanism for loss of p53 function is through mutation, however, about half of all cancers retain the ability to express normal p53 protein deregulated by other mechanisms [32]. In this regard, HIPK2 is often inactivated in tumors by multiple mechanism such as gene downregulation [33,34], gene mutation [35], protein mislocalization [36] as well as by protein degradation through MDM2 [37] or hypoxia-induced factors [38], impairing the cellular response to drug and supporting tumor progression. In addition, the malignant phenotype was shown in HIPK2 heterozygous mice [39], suggesting that HIPK2 may restrain tumor development and progression by acting on multiple different but sometime interconnected pathways. However, we recently reported that hypoxic-induced HIPK2 inhibition can be completely restored by zinc supplementation that reactivates p53 apoptotic response to drug [40], hypothesizing that HIPK2 gene or protein inactivation can make a big difference in rescuing p53 activity and therefore tumor response to drug in terms of therapeutical strategies. Therefore, our studies not only provide a view of the multiple levels at which HIPK2 might fine-tune the transcriptional activity of p53 in tumor cells subjected to genotoxic stress, including the novel acetylation/deacetylation balance and Sirt1 regulation, but also may be relevant for designing new strategies for treatment of tumors with non-functional wtp53 and/or HIPK2.

## Conclusion

As the knowledge of p53 activation by posttranslational modification and protein/protein interactions grows the challenge is to translate all this information into efficient anticancer therapies. Our data suggest that a decrease of HIPK2 levels is indicative of negative response to drug therapy of cancer cells with wtp53 as it deeply affects p53 posttranslational modifications and therefore p53 transcriptional activity. Many studies have recently focused on pharmacological strategies aimed at restoring p53 transcriptional function to look for new therapeutic approaches to be translating in the clinic [41]. Therefore, our results may represent an improvement to cancer therapy by combining pharmacological or natural agents with standard therapies to be applied in the clinic for the potential reactivation of wtp53 function in tumors.

## Methods

### Cell cultures and treatments

RKO, MCF7, and H1299 (p53/null) were cultured in RPMI-1640 (GIBCO-Invitrogen) supplemented with 10% fetal bovine serum (GIBCO-Invitrogen), glutamine and antibiotics. Doxycycline (Dox)-inducible MCF7 (MCF7indsi/HIPK2) cell line expressing HIPK2-interference has been described [23]. For inducible HIPK2 knockdown Dox (1 μg/mL) was added to MCF7indsi/HIPK2 cells every 3 days until HIPK2 knockdown was successfully reached (usually in about 5 days). After HIPK2 knockdown was reached, cells were cultured without Dox for additional 5 days before harvesting and processing for RT-PCR and ChIP analyses. Stable HIPK2 interference was obtained in RKO and H1299 cells after transfection of pSuper-control and pSuper-HIPK2 vectors and selected as mix population, as reported [6]. RKO cells were stably transfected with the p53AIP1-luc reporter along with pBabe-puro vector (1:10 molar/ratio), as reported [42]. Twenty-four hours after transfection puromicin (2 μg/ml) was added to the cell culture medium for selection. Dox (1 μg/mL), Adriamycin (ADR, 2 μg/ml), ZnCl_2 _(150 μM), nicotinamide (Nic, 5 mM) and Trichostatin A (TSA, 100 nM) all from SIGMA (St. Louis, MO, USA), were used as indicated.

### Plasmids and mutagenesis

The following expression vectors and reporter plasmids were used: pSuper and pSuper-HIPK2 [6], HIPK2-Flag [5] p300-Flag (kindly provided by M. Fanciulli, Regina Elena Cancer Institute, Rome, Italy), pcDNA3-wtp53, K382R (lysines 381 and 382 mutated to arginine) and p53Q373 (lysines 370, 372, and 373 mutated to glutamine), and A46Q373 (serine 46 mutated to alanine in Q373 vector) were generated by using PCR-based site-directed mutagenesis (Qiagen, Crawley, UK), p53AIP1-luc (kindly provided by H. Arakawa, National Cancer Center, Tokyo, Japan), Noxa-luc (kindly provided by T. Shibue, Graduate School of Medicine, University of Tokyo, Japan), p21-luc (kindly provided by B. Vogelstein, Johns Hopkins University, Baltimore, MD, USA), and GADD45-luc (kindly provided by K. Sabapathy, National Cancer Centre, Singapore).

### Transfection and transactivation assay

Transient or stable transfection were carried using the calcium phosphate methods or the cationic polymer LipofectaminePlus reagent (Invitrogen, Carlsbad, CA, USA) according to the manufacturer's instructions. The amount of plasmid DNA in each sample was equalized by supplementing with empty vector.

For transactivation assay cells were co-transfected with the reported expression vectors along with the luciferase reporter gene driven by p53-target promoters. Transfection efficiency was normalized with the use of a co-transfected β-galactosidase (β-gal) plasmid. Luciferase activity was assayed on whole cell extract and the luciferase values were normalized to β-galactosidase activity and protein content. At least three independent experiments were performed in duplicate.

### Chromatin Immunoprecipitation (ChIP) and Re-ChIP Assays

ChIP and Re-ChIP assays were performed as described [43]. Protein complexes were cross-linked to DNA in living cells by adding formaldehyde directly to the cell culture medium at 1% final concentration. Chromatin extracts containing DNA fragments with an average size of 500 bp were incubated overnight at 4°C with milk shaking using polyclonal anti-p53 (FL393, Santa Cruz Biotechnology), antiserum anti-acetyl-p53 (Lys373&382) (Upstate), polyclonal anti-acetyl-p53 (Lys382) (Cell Signaling), monoclonal anti-p300 CT (clone RW128) (Upstate), and antiserum anti-PAN-acetylated Histone 4 (ac-H4, Upstate) antibodies. DNA-protein complexes were recovered with protein G Agarose (Pierce). PCR was performed with HOT-MASTER Taq (Eppendorf) using 2 μL of immuniprecipitated DNA and promoter-specific primers for for the p53 target promoters. In each experiment, the linearity of the signal was insured by amplification of increasing amounts of template DNA. Immunoprecipitation with non-specific immunoglobulins (IgG; Santa Cruz Biotechnology) was performed as negative controls. The amount of precipitated chromatin measured in each PCR was normalized with the amount of chromatin present in the input of each immunoprecipitation. PCR products were run on a 2% agarose gel and visualized by ethidium bromide staining using UV light.

### RNA Extraction and Reverse-Transcription (RT) PCR

Total RNA was extracted with TRIzol (Invitrogen) following the manufacturer's instructions. The first strand cDNA was synthesized by reverse-transcribing 5 μg of mRNA with Moloney murine leukaemia virus reverse transcriptase kit and random primers (Applied Biosystems). Semiquantitative RT-PCR was carried out by using Hot-Master Taq (Eppendorf) with 2 μl cDNA reaction and genes specific oligonucleotides under conditions of linear amplification. DNA products were run on 2% agarose gel and visualized by ethidium bromide staining using UV light. Data presented are representative of at least three independent experiments.

### TUNEL assay

For TUNEL assay, 4 × 10^4 ^cells were spun on a slide by cytocentrifugation and subsequently fixed in 4% paraformaldehyde for 30 min at room temperature. After rinsing with PBS the samples were permeabilized in a solution of 0.1% Triton X-100 in sodium citrate for 2 min. Samples, washed with PBS, were then incubated in the TUNEL reaction mix for 1 h at 37°C, according to the manufacturer's instructions (Roche, Germany). Cells were counter-stained with Hoechst 33342 before analysis with a fluorescent microscope (Zeiss). Standard deviations of three independent experiments were indicated.

### Western Blotting and p53 conformational immunoprecipitation

Total cell extracts and subcellular fractionations were carried out as [34]. Proteins were then separated by 4-10% gradient SDS-PAGE (BioRad) and blotted onto nitrocellulose membrabe (BioRad). The membranes were probed with a primary antibody followed by horseradish-peroxidase conjugated secondary antibody. Immunoreactivity was detected with the ECL-chemoluminescence reaction kit (Amersham). The antibodies used were: monoclonal anti-p53 (DO1), polyclonal anti-p53 (FL393), polyclonal anti-Bax, polyclonal anti-p21 (Santa Cruz Biotechnology), polyclonal anti-p53Ser46 (Cell Signaling), polyclonal anti-acetyl-p53 (Lys382) (Cell Signaling), monoclonal anti-p300 CT (clone RW128) (Upstate), monoclonal anti-poly(ADP-ribose) polymerase (PARP, BD PharMingen), monoclonal anti-tubulin (Immunological Sciences), and monoclonal anti Hsp70 (Stressgene).

Conformational immunoprecipitation was essentially performed as [22]. Briefly, 150-250 mg of total cell extracts were immunoprecipitated with conformation-specific monoclonal antibody PAb240 (mutant specific) (Calbiochem). Immunocomplexes were separated by 10% SDS-PAGE and immunoblotting was performed with rabbit anti-p53 antibody (FL393) (Santa Cruz). Immunoreactivity was detected with the ECL-chemoluminescence reaction kit (Amersham).

### Statistics

All experiment unless indicated were performed at least three times. All experimental results were expressed as the arithmetic mean and standard deviation (s.d.) of measurements was shown. Student's *t*-test was used for statistical significance of the differences between treatment groups. Statistical analysis was performed using analysis of variance at 5% (p < 0.05) or 1% (p < 0.01).

## Abbreviations

ADR: adriamycin; ChIP: chromatin immunoprecipitation; Dox: Doxycyclin; HAT: histone acetyltransferases; Nic: nicotinamide; RT-PCR: reverse-transcriptase-polymerase chain reaction; TSA: trichostatin A.

## Competing interests

The authors declare that they have no competing interests.

## Authors' contributions

RP and LN performed the experiments. GDO conceived the experimental design and wrote the paper. DG, GR and AS revised critically the paper. All authors read and approved the final manuscript.

## Supplementary Material

Additional file 1 Table S1 - selected p53-target genes unregulated in RKO control (siRNA) and HIPK2 depleted (siHIPK2) in response to ADR Click here for file
