# Supplementary figures and images for: HIPK2 modulates p53 activity towards pro-apoptotic transcription
Source: Mol Cancer. 2009 Oct 14;8:85. doi: 10.1186/1476-4598-8-85 (PMC2768676; doi:10.1186/1476-4598-8-85)

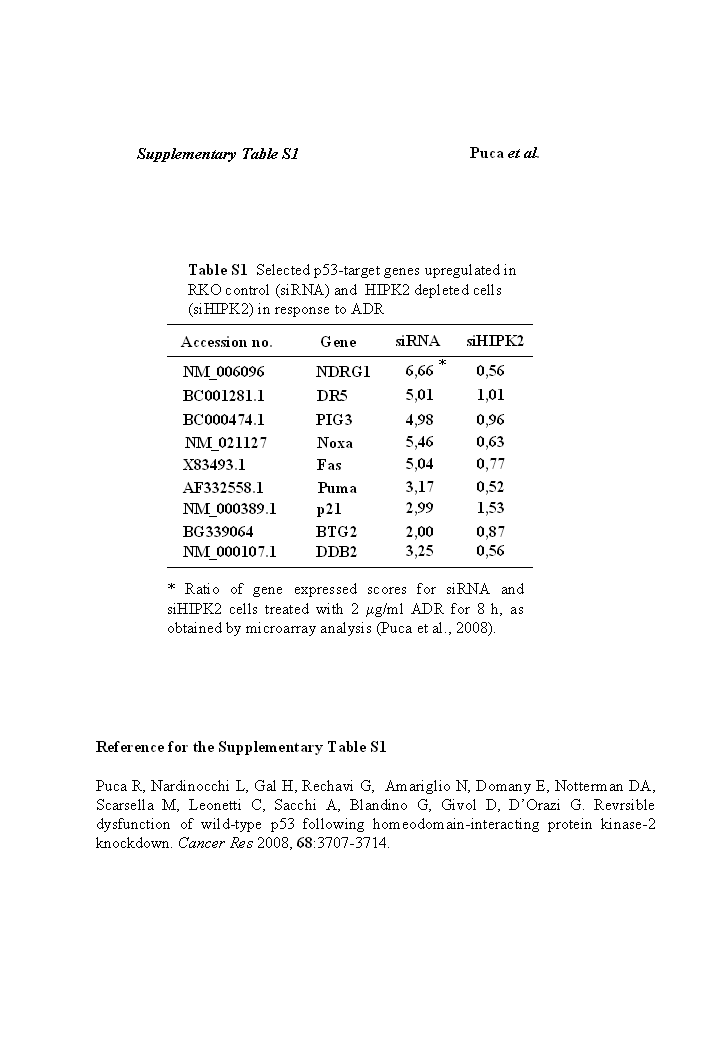

Supplement: Additional file 1 — Table S1 - selected p53-target genes unregulated in RKO control (siRNA) and HIPK2 depleted (siHIPK2) in response to ADR [file 1476-4598-8-85-S1.TIFF]
